# Treadmill Exercise Induced Functional Recovery after Peripheral Nerve Repair Is Associated with Increased Levels of Neurotrophic Factors

**DOI:** 10.1371/journal.pone.0090245

**Published:** 2014-03-11

**Authors:** Jae-Sung Park, Ahmet Höke

**Affiliations:** Departments of Neurology and Neuroscience, The Johns Hopkins University School of Medicine, Baltimore, Maryland, United States of America; University of Edinburgh, United Kingdom

## Abstract

Benefits of exercise on nerve regeneration and functional recovery have been reported in both central and peripheral nervous system disease models. However, underlying molecular mechanisms of enhanced regeneration and improved functional outcomes are less understood. We used a peripheral nerve regeneration model that has a good correlation between functional outcomes and number of motor axons that regenerate to evaluate the impact of treadmill exercise. In this model, the median nerve was transected and repaired while the ulnar nerve was transected and prevented from regeneration. Daily treadmill exercise resulted in faster recovery of the forelimb grip function as evaluated by grip power and inverted holding test. Daily exercise also resulted in better regeneration as evaluated by recovery of compound motor action potentials, higher number of axons in the median nerve and larger myofiber size in target muscles. Furthermore, these observations correlated with higher levels of neurotrophic factors, glial derived neurotrophic factor (GDNF), brain derived neurotrophic factor (BDNF) and insulin-like growth factor-1 (IGF-1), in serum, nerve and muscle suggesting that increase in muscle derived neurotrophic factors may be responsible for improved regeneration.

## Introduction

Physical activity and exercise interventions are used to promote general health, prevent and delay development of the chronic disease and combat effects of aging [1], [2]. In addition, various exercise interventions have been used to improve motor function after spinal cord injury both in animal models and in clinical practice [3]–[6]. Other neurological diseases where exercise has been shown to be effective include Parkinson's disease [7], Alzheimer's disease [8] and diabetic neuropathy [9] among many others. The impact of exercise on peripheral nerve regeneration has attracted relatively little attention [10].

Unlike central nervous system injury, when peripheral axons are injured there is a robust regenerative response that results in good functional outcomes with distal nerve lesions. However, proximal nerve lesions result in poor recovery partly due to slow rate of regeneration and chronic denervation changes that take place in the distal segments of the nerve and in the target muscle [11]. Strategies that enhance axonal growth would have a beneficial effect on peripheral nerve regeneration.

Since exercise has been shown to enhance neurite outgrowth in dorsal root ganglion neurons, acutely isolated from exercised animals, [12] and that this effect was neurotrophic factor dependent, we examined the effect of exercise on peripheral nerve regeneration using a comprehensive battery of outcome tools and measured the levels of muscle derived neurotrophic factors. We utilized a median nerve repair model of peripheral nerve regeneration. In this model, the median nerve is transected and repaired in the upper arm while the ulnar nerve is completely resected out preventing contribution of ulnar nerve to handgrip function. This model shows a better linear correlation between functional evaluations (handgrip strength and electrophysiology) and number of axons that regenerate in the median nerve. Using this model, we show that daily treadmill exercise affords faster functional recovery and close correlation with increased levels of neurotrophic factors in muscles, sera and distal nerves.

## Materials and Methods

### Animals

This study was carried out in accordance with the recommendations in the Guide for the Care and Use of Laboratory Animals of the National Institutes of Health. The protocol was approved by the Johns Hopkins University Animal Care and Use Committee. All surgical procedures were conducted under sterile conditions and all efforts were made to minimize suffering. Adult male wild type mice on a C57Bl/6J background, purchased from Jackson Laboratories, were used. The animals were 6–8 weeks old and weighed between 20∼30 grams. Animals were randomized to three groups: Control group (Con), Nerve repair without Exercise group (No Ex) and Nerve repair with Exercise group (Ex). Each group consisted of 8 animals.

### Median nerve repair model

All surgeries were done under deep inhalation anesthesia with isoflurane in aseptic conditions. Briefly, median and ulnar nerves were exposed in the upper forelimb in anesthetized animals. The median nerve was repaired by immediately suturing both ends with 10-0 suture material. The ulnar nerve was tied with 8-0 silk and deflected to biceps muscle to prevent regeneration. The surgical site was closed with sterile staples and the animals were returned to regular housing with adequate analgesics. The next day after surgery, adequacy of the median nerve transection was confirmed with nerve conduction studies in which the median nerve was stimulated above the repair site and compound motor action potential (CMAP) was recorded in the hand muscles under inhalation anesthesia. Throughout the study, the animals were monitored for development of autotomy.

### Low-intensity aerobic treadmill running program

All mice were placed on the treadmill for 30 minutes every day for a week prior to surgery to acclimatize them to treadmill machine. After the operation, all mice had three days of rest and the exercise program started on day 4 after the surgical repair. Exercise program consisted of 60 minutes of continuous running at a 10 m/min speed with a 5 minute warm up and 5 minutes of cool down (at a speed of 6 m/min) with no incline. This was done 5 days a week for six weeks as previously described [10]. Mice subjected to median nerve transection were able to run at 10 m/min for 1 h continuously beginning three days after surgery, despite the loss of unilateral forelimb grip strength. Mice in the nerve repair without exercise group were kept in their cages and did not receive treadmill exercise. Mice in the control group remained caged for six weeks after sham surgery where the skin was opened and the median and ulnar nerves were exposed but not transected. These mice did not receive treadmill training.

### Functional measurements of muscle power

To assess functional recovery, grip strength tests were carried out once a week from pre-surgery to end of experiment. Grip strength test was performed using the Chatillon force measurement device (Ametek, Largo, FL). The mouse was held by the tail and allowed to grasp the bar on the device. As the experimenter pulled the tail at a 45-degree angle, the maximum force generated was recorded. This measurement was done 3 times for each animal and average score was recorded.

Functional recovery was also evaluated using wire grip holding test once a week starting at baseline before surgery until the end of the experiment. In this assay, each mouse was placed at the top of the wire grid and allowed to accommodate to this environment for 3–5 seconds before the grid was inverted and held approximately at 30 cm height from the bench. Each of these holding periods began with all four paws of the mouse grasping the wire grid. The wire grid holding time is defined as the amount of time that it takes the mouse to fall from the inverted screen and was measured visually with a stopwatch. In each session, the procedure was repeated three times with approximately 5 min or more between each assessment of holding time.

### Evoked nerve conduction studies

Evoked nerve conduction studies were performed on the median nerve using techniques standard for our laboratory. After the animals were anesthetized with isoflurane, recording electrodes were placed over the median innervated muscles in the ventral forearm. The median nerve was stimulated proximal to the injury at the deltoid tuberosity with a bipolar subdermal needle electrode (CareFusion, Middleton, WI) and recording was carried out with PowerLab (AD Instruments, Colorado Springs, CO).

### Nerve morphometry

After 6 weeks of treadmill exercise, animals were anesthetized with isoflurane and decapitated. For morphological evaluation, a 2-mm segment of the median nerve at 3–5 mm distal to the cut and repair site was rapidly excised, fixed in a solution of 4% paraformaldehyde and 3% glutaraldehyde for two days, and then transferred into Sorensen's phosphate buffer (0.1M) for further processing as described previously [13]. Briefly, the nerves were postfixed in osmium tetroxide, embedded in plastic, sectioned at 1 µm using an Ultracut E microtome (Reichert Technologies, Depew, NJ), and stained with toluidine blue (1% toluidine blue in 1% sodium tetraborate). Digital images of the median nerve were taken using an unbiased sampling method of non-overlapping regions of the whole cross section of the median nerve. Total number of myelinated axons per cross-section of each regenerated nerve was quantified using Image J software. In addition, the G ratio, which is defined as the ratio of axon diameter to the total diameter of the nerve fiber, was calculated. For each sample, at least 200 myelinated axons were measured and the average was counted as n = 1.

### Immunohistochemistry

Forearm extrinsic finger flexor muscles innervated by the median nerve were harvested at the end of the study, stretched on a small piece of cardboard with pins, fixed in 4% paraformaldehyde and then frozen. Tissue imbedded in OCT was sectioned with a cryostat (Microm HM 550) into 10 µm sections. Slides were stained by blocking in 5% BSA/PBS then incubated over night with primary antibody against laminin-γ1 (catalogue number: MAB1920, Chemicon, Billerica, MA) in 1% BSA/PBS at 4°C. Slides were washed 3 times for 5 minutes with 1% BSA/PBS, incubated for 90 minutes at room temperature with appropriate secondary antibody (Fluorescein Horse Anti-Mouse IgG Antibody, catalogue number Fl-2000, Vector Laboratories, Burlingame, CA), washed again 3 times for 5 minutes with 1% BSA/PBS, and then mounted with hard set mounting media with Dapi (catalogue number: H-1500, Vector Laboratories, Burlingame, CA). Immunofluorescent pictures were taken with an Eclipse i80 microscope (Nikon) at 10× magnification for the purpose of myofiber measurement analysis. Myofiber size was determined by measuring the minimum ferrets diameter of 700–1000 fibers for 3–5 mice per treatment group using Nikon NS elements 2.0 software. Average from each animal was counted as n = 1. Representative pictures of myofiber size were taken at 20× magnification.

### Neurotrophic factor (GDNF, BDNF, IGF-1) protein measurements

Total blood was collected from the exposed heart at the time of decapitation of deeply anesthetized animals and incubated at room temperature for 30 minutes. Then serum was obtained by centrifuging the total blood at 2000 rpm at 4°C for 15 minutes and stored at −80°C until analysis. Tissue Protein Extraction Regent (T-PER) was used for protein extraction from distal median nerve and median nerve innervated forelimb muscle. Protease inhibitor (Thermo Scientific Halt Protease Inhibitor Cocktail, EDTA-free) was added to the T-PER reagent just before use. Twenty ml of T-PER reagent per 1 gram of tissue was added and tissues were individually homogenized. Samples were then centrifuged at 10,000×g for 5 minutes to pellet cellular and tissue debris. Supernatant was collected and protein levels of glial derived neurotrophic factor (GDNF), brain derived neurotrophic factor (BDNF) and insulin-like growth factor-1 (IGF-1), were measured using ELISA according to manufacturer's protocols (Catalogue numbers: GDNF-G7620 and BDNF-G7610, Promega, Madison, WI and catalogue number: ab100695 IGF-1 mouse ELISA kit, Abcam, Cambridge, MA).

### Statistical analysis

Statistical analysis was done using analysis of variance with correction for multiple comparisons (critical α level set at p = 0.005).

## Results

### Exercise induced recovery of strength and electrophysiological parameters

**Figure 1:**
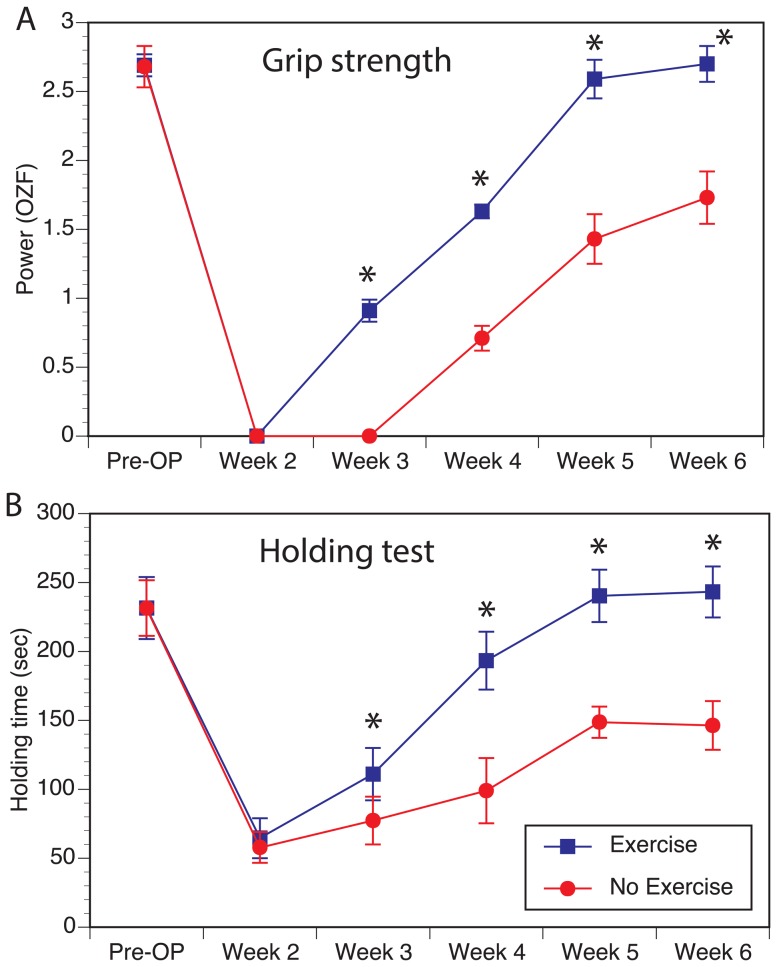
Effect of exercise on functional outcomes. Exercise improved both grip strength (**A**) and time on the inverted holding test (**B**) in mice with median nerve repair over 6 weeks. (* Denotes statistically significant difference, P>0.05).

Daily regular treadmill exercise resulted in faster and more complete recovery of both grip strength and holding ability in mice that underwent unilateral median nerve repair (Figure 1). Due to complete resection of the ulnar nerve, the no-exercise group did not have full recovery even at the 6-week time point.

**Figure 2:**
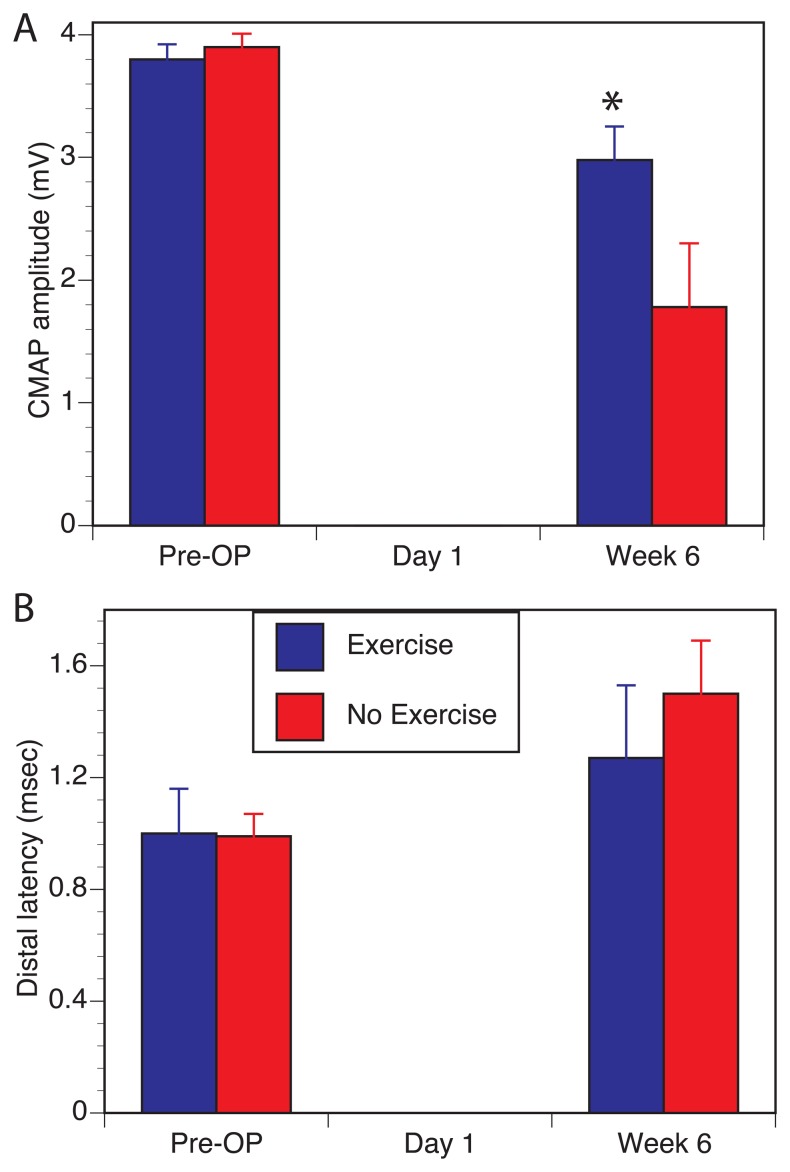
Effect of exercise on evoked motor response in nerve conduction study. Exercise improved both the amplitude of the evoked CMAP response (**A**) and distal latency (**B**) 6 weeks after median nerve repair. (* Denotes statistically significant difference, P>0.05).

In parallel with the functional recovery in grip strength, we also observed a better recovery of evoked motor responses recorded in distal median nerve innervated forearm muscles (Figure 2). Evoked motor responses were done at baseline, day 1 after surgery to confirm the completeness of the nerve transection and at the endpoint of 6 weeks. As seen in figure 2A, the amplitude of the CMAP was greater in the exercise group compared to no-exercise group. Similarly, the distal latency was shorter indicating better myelination of the regenerating fibers (Figure 2B).

### Morphological evidence of exercise induced nerve regeneration

**Figure 3:**
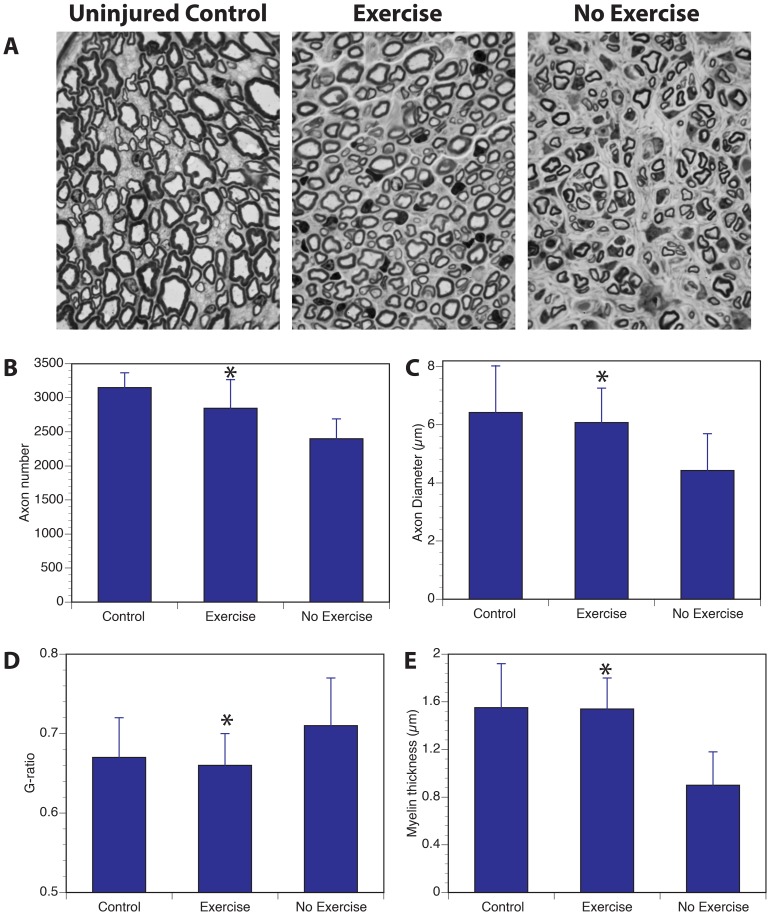
Impact of exercise on nerve morphometry. Exercise resulted in improved nerve regeneration as shown cross sections of distal median nerve in uninjured control nerve, regenerated nerve with exercise and regenerated nerve with no-exercise (**A**). Quantitation of the nerve morphometry showed higher number of axons (**B**) with larger diameter (**C**) and thicker myelin (**E**). This resulted in normalization of the g-ratio (**D**). (* Denotes statistically significant difference compared to no-exercise group, P>0.05).

In order to find morphological correlates of enhanced functional and electrophysiological recovery, we carried out both nerve and muscle morphometry in distal median nerves and median nerve innervated forearm muscles, respectively. As shown in figure 3, the exercised group had higher number of regenerated axons and these axons were larger in diameter with more mature myelination as measured by G-ratio and myelin thickness.

**Figure 4:**
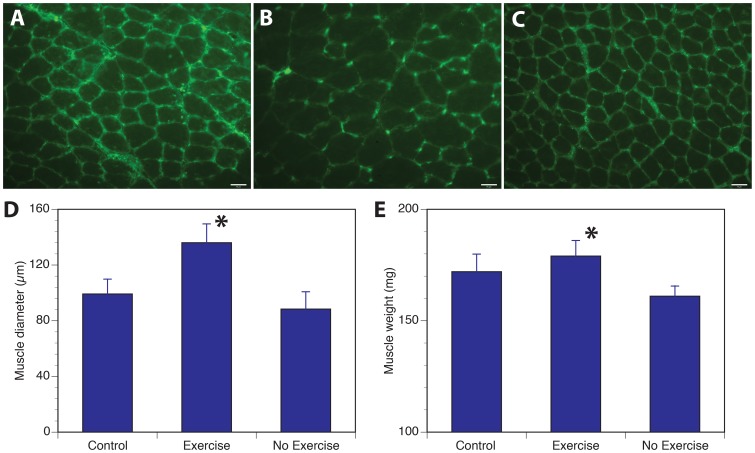
Effect of exercise on muscle mass. Exercise resulted in larger myofiber size as shown in (**B**) compared to uninjured control (**A**) or no-exercise group (**C**). This is quantified in (**D**). There was also a difference in total muscle weight between the exercise and no-exercise groups (**E**). (* Denotes statistically significant difference compared to no-exercise group, P>0.05).

In terms of the impact of nerve injury and exercise on median nerve-innervated forearm flexor muscles, there was mild atrophy in the no-exercise group compared to the uninjured control mice but the exercised group resulted in clear muscle hypertrophy, even compared to the uninjured control group (Figure 4). The laminin staining was less prominent in the exercise group, even though the staining protocols were the same across all groups.

### Treadmill exercise enhances expression of neurotrophic factors in serum, nerve and muscle

**Figure 5:**
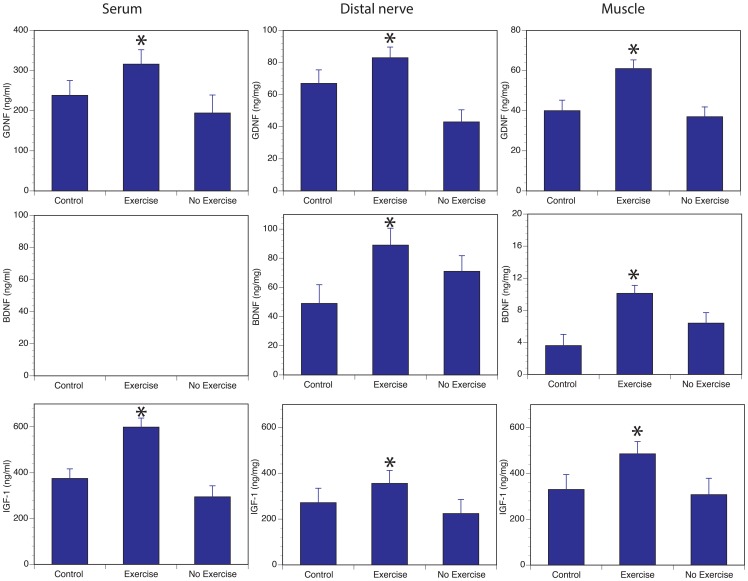
Effect of exercise on neurotrophic factor levels. Tissue and serum levels of GDNF, IGF-1 and BDNF were measured at 6 weeks after nerve repair. In all cases, except serum BDNF, exercised group had higher levels of all three neurotrophic factors in muscle, serum and distal nerve as measured by ELISA. Control is uninjured animals with no exercise program. Exercise and no exercise groups denote animals undergoing median nerve repair and regeneration. (* Denotes statistically significant difference compared to no-exercise group, P>0.05).

We hypothesized that one potential way as how exercise could exert a remote effect on nerve regeneration was through an increase in neurotrophic factors. As seen in figure 5, levels of GDNF, BDNF and IGF-1 were all higher in serum, distal nerve and forearm muscles at 6 weeks in the exercise group compared to the no exercise group (with the exception of serum BDNF levels which were below the detection limit of the ELISA assay).

## Discussion

In recent years, there has been a growing interest in evaluating the remote effects of exercise on various neurological conditions, including peripheral [10], [14], [15] and central [16]–[18] nervous system injury (reviewed in [19], [20]). Here we report the effects of regular treadmill exercise on peripheral nerve regeneration in a model of peripheral nerve repair that shows good correlation between the numbers of axons that regenerate and behavioral functional outcome measures used. Furthermore, we show that improved peripheral nerve regeneration correlates with an increase in neurotrophic factor expression at the protein level in the muscle, serum and distal nerve.

Similar to previous reports examining the effect of exercise on peripheral axonal regeneration using the sciatic nerve injury model, we also observed enhanced regeneration using a variety of outcome measures when median nerve injury was repaired and mice were exercised for about an hour 5 days/week. This finding confirms previous reports and extends the beneficial effect of regular aerobic exercise to another nerve injury and repair model. In previous reports, the investigators had observed a gender difference in terms of the type of exercise needed to see the beneficial effect of exercise [21]. We elected to use a single gender, i.e. male mice and an exercise regimen that had been shown to be beneficial in male mice before. It is unclear if the beneficial effects we observed in the median nerve repair extend to the female mice and if so, with what type of exercise regimen. This issue needs to be addressed extensively, as clinical translation of the role of exercise on improving neurological outcomes requires determining the optimum exercise regimens for both genders.

Unlike previous reports, we utilized a multitude of outcome measures and demonstrated the effect of treadmill exercise on all modalities including functional behavioral improvement in two different tests that evaluate motor recovery, electrophysiological measurement that evaluates motor reinnervation and histological measurement of regenerated myelinated axons in a mixed nerve. Of the two behavioral outcome measures, the grip strength recovery was accelerated by about a week and had shown evidence of recovery as early as 3 weeks after nerve repair. It is interesting to note that there was no full recovery for up to 6 weeks in the no-exercise group, likely a reflection that part of the lack of recovery is due to loss of ulnar-innervated forelimb flexors. The fuller recovery seen in the exercise group may also point to compensation of ulnar loss by hypertrophy seen in the median innervated muscles (Figure 4). However, this alone could not explain the near-full recovery in the exercise group because the measurement of CMAP amplitudes (Figure 2) and higher number of regenerated axons (Figure 3) in the exercise group points to contribution from regeneration of more motor axons in the repaired median nerve. Exercise also induced better myelination of the regenerated axons as indicated by reduced distal latency of the evoked motor response as well as more normalized g-ratios in the nerve morphometry studies. What is unclear from our studies is that the effect of exercise on denervated and reinnervated muscle may be different that the effects of exercise on an intact muscle. As seen in Figure 4, muscles in the exercise group had two populations of fibers with different sizes compared to the non-exercise group suggesting that muscle hypertrophy may be due to effects of exercise only on a sub-population of myofibers. Future studies looking at the impact of exercise on nerve regeneration need to consider the direct effect of exercise on muscle and functional outcomes used.

In order to explain the remote effects of exercise on regenerating axons, we evaluated the expression of 3 known neurotrophic factors in the muscle, serum and distal nerve. As expected, GDNF, BDNF and IGF-1 protein levels were all increased in exercised muscle; these findings are similar to previously published studies [22]–[24]. However, we also showed that exercise results in increase in serum levels of GDNF and IGF-1. Despite multiple attempts we were unable to detect BDNF in the serum in these mice. It is unclear if this was due to a technical issue with the ELISA protocols or true lack of BDNF in high enough quantities in the serum of mice. Nevertheless, others have shown that there is increase in BDNF in both neurons and Schwann cells with exercise and these increases play a role in exercise induced enhanced peripheral nerve regeneration [25].

It is interesting to note that we also observed higher levels of GDNF, BDNF and IGF-1 in the distal nerve of exercised mice compared to no-exercise group. Obviously this could be due to increased levels in serum (at least for GDNF and IGF-1), but another likely explanation is that exercise may have improved blood flow overall in the whole animal and resulted in improved activation of Schwann cells [25], [26]. Clearly exercise has been shown to result in neovascularization and increased blood flow and is likely to be due to direct result of increase in expression of angiogenesis related genes [27]

One limitation of our study is that we looked at the neurotrophic factor expression at the end of our study, i.e. 6 weeks, at a time when reinnervation and recovery were well underway. It is possible that there was a bigger impact of exercise on neurotrophic factor expression at earlier time points. Nevertheless, presence of increased expression of all three neurotrophic factors in the distal nerve at 6 weeks suggests the effects of exercise is likely to be long-lasting and maintained even after nerve regeneration has taken place.

Despite our attempt at demonstrating a correlation between increased neurotrophic factor expression and enhanced peripheral nerve regeneration with exercise, we still do not fully know how exercise influences remote events such as nerve regeneration or neuroprotection in mouse models of neurodegenerative diseases. One relatively straightforward explanation maybe improved blood flow and enhanced overall rate of metabolism. Another possibility is that muscle releases, a yet unknown poietic factor, into the blood, which then acts at remote sites such as brain and nerves to induce regenerative or cell defense mechanisms. The diverse effects of exercise may be mediated by multiple muscle derived factors [28]. The relative ease of the median nerve repair model as well as the robustness of the outcome measures used in this study will help us tease out the underlying molecular mechanisms of regenerative effects of regular aerobic exercise. In the meantime, there is little downside to recommending a regular aerobic exercise to patients undergoing peripheral nerve repair after traumatic injury or patients with peripheral neuropathies.
